# The Institutional Worker

**Published:** 1918-07-27

**Authors:** 


THE
### INSTITUTIONAL WORKER
**Being a Special Supplement to "The Hospital"**

### OUR BUREAU OF INFORMATION.

**Rules for Correspondents.**

1. Every letter must be accompanied by the coupon to be cut from the back cover (inside page) of The Hospital, current issue, and must contain the name and address of the correspondent with pseudonym for publication if desired. Replies by post cannot be given save under exceptional circumstances at the Editor's discretion.

2. Letters from Approved Homes in reply to special needs published in the Bureau should state terms and full particulars, and be sent prepaid under cover to the Editor of the Bureau with name written across coupon for identification.

3. Proprietors of Homes which have not yet been entered on the List of Approved Homes, but have spare accommodation likely to suit special needs, are invited to write for an application form for registration. The fee for registration, which includes two announcements of the Home in the Bureau and other privileges, is 10s.

4. All communications to be addressed to the Editor of The Hospital, 28 Southampton Street, Strand, London, W.C. 2, and marked "Bureau of Information."

### INSTITUTION FACTS AND FIGURES.

### The Steward's Department of a Large Hospital.

### II.—THE STORES AND STOREKEEPING.

In these days, when prices vary so considerably and the cost of goods rapidly increases, it is essential that the steward should keep in touch with market prices. He is fortunate if he has a friend—a professional buyer—who will let him accompany him on his rounds of the market. The experience gained in this way should prove most valuable. If the steward has the interests of the hospital at heart he will be on the look-out for every opportunity of buying at a reasonable price goods that will not deteriorate by being kept in stock. Nowadays, with fluctuating markets and prices jumping up with leaps and bounds, it is more important than ever to save the expenditure of the hospital in every way possible.

#### Foresight in Buying.

Frequently the manager of a firm which does business with the institution will advise the steward as to the advisability of getting in a good supply of certain articles likely to become scarce or very expensive. The managers generally know, and if they have been treated well by the hospital they will only be too glad to do the hospital a good turn if an opportunity comes their way, which advice, coming from a reputable and reliable firm, the steward may well accept. For instance, the manager of the soap firm with which the hospital may have contracted off and on for years will advise the steward to get in a good supply of a certain kind of ordinary soap much used in the hospital. He will know that it is going to be very expensive and difficult to get. The steward, when he has satisfied himself that it would be a good thing to lay in a stock of such soap or any other article, will order accordingly, and in time to come will probably be very grateful for the advice he has had on the matter.

#### The Store-Rooms.

To be able to lay in a good stock of goods, however, it is very necessary to have adequate store room. It is not sufficient only to have accommodation for the weekly or monthly requirements; there must be plenty of room for storing the goods if the steward is to use his opportunities of saving the hospital money.

There should be a large room, with an office for the storekeeper attached. The goods would be delivered and checked in this room, as it is not desirable that people from outside the department should have access to the stores. The staff of men and boys employed here should be reliable and trustworthy, as they will have many temptations in handling the goods.

It is desirable to have a good milk-room, tiled, and on the cool side of the building, but at the same time in a position facilitating easy delivery. Other stores required would be for bread, grocery, crockery, stationery, hardware, mattress-room, beer-room, a place for empty boxes and bottles, and a porter's room.

#### Method of Distribution.

Milk, bread, beer, and special articles of grocery are delivered to the wards daily. These goods will be ordered by the sister through the patients' diet-books, and after the summary from the diet-books has been worked out by the steward's clerks the storekeeper will be notified by the steward as to what quantities he must deliver to the wards.

A day in the week will be set apart for giving out crockery, another day for stationery and hardware, and in this way the giving out of stores will be more or less evenly distributed. The heads of departments and sisters must sign a voucher when the stores are delivered to them, otherwise there would be no check as to whether the articles ordered had been received.

#### Book-Keeping.

The storekeeper should enter in his book the quantities of each class of goods he receives and the goods he gives out, and these quantities when entered must correspond with his order-books. At the end of the day or week he should enter up his book so that he knows exactly what stock he holds. His book will then be checked with the invoices by the steward or his clerks before the invoices are passed on for payment.

Before the invoices are passed on to the accountant, however, the amounts should be entered in a ledger in the steward's office under the headings of the different departments the goods have been delivered to. It is quite an easy matter by this method of book-keeping readily to work out the cost of a department. Periodically the steward will take stock of the stores to check it and see if it corresponds with the store-book.

#### Checking of Waste.

Waste bread should not be thrown away, but should be collected each day in baskets and returned to the stores. The baskets should be labelled with the name of the ward, for in this way it is easy to trace any excessive waste in a ward. The bread should be sold eventually for the manufacture of dog-biscuits or other such things.

The refuse from the plates should be collected daily in pails and removed from the wards, not for destruction but to sell as pig-wash.

As the meat is all carved on hot plates in the kitchen and sent to the wards in hot tins there should be very little waste meat over from the wards. Any meat over in the kitchen will be used up as far as possible, and that which is no use at all will be put with the wash and sold.

A careful steward will do all he can to prevent waste, but that which is unavoidable he will sell for the good of the hospital.

*(To be continued.)*

☛ **The first article appeared July 20.**

### JULY QUESTIONS.

### 1. War Vacancies.

**Give model "Terms of Appointment" applicable for the substitute of a hospital's administrative official who has been called to the Colours. Provision to be made for the possible return to duty of the official previous to the termination of the war and the filling of the appointment in case of the non-return of the official concerned. Describe fully any experiences in this connection that may prove of value to hospital managers generally.**

### 2. Bedroom Suppers for Nurses Off Duty.

**In those hospitals having no kitchen in the Nurses' Home what arrangements can be made whereby the sisters and nurses who are off duty in the evening may have the comfort of supper in their bedrooms? What method may be adopted in order to avoid the dining-room maid being faced by a more or less depleted pantry when she wishes to lay breakfast? (The difficulty seems to be not so much as regards the food as the crockery.)**

### RULES.

The following rules must be observed:—

1. Contributions must be written on one side of the paper. Brevity and terseness are desirable features. The MSS. must bear the name and address of the sender and be accompanied by coupon to be cut from the back cover (inside page) of the current issue of THE HOSPITAL. A pseudonym must be chosen if the name is not to be published.

2. Contributions must be addressed to the Editor of THE HOSPITAL, Institutional Worker Supplement, 28 & 29 Southampton Street, Strand, London, W.C. 2, should reach him before the end of the current month, and be marked in the left-hand corner "Facts and Figures."

**A minimum payment of Five Shillings will be made for each published answer.**

### QUESTIONS INVITED.

In connection with our Question Box, we offer every month five shillings each for the two best questions which are sent in for consideration. The questions may be on any institutional subject and concern any department, from the secretary's to the porter's, or the matron's to the domestic staff; the one essential is that they must be practical and deal with points that have a definite relation to hospital or institutional administration, upkeep, finance, or management. Points relating to artificers' work, housekeeping, the laundry, out-patients, and other practical matters will be welcome. It is hoped that thus, when difficult or doubtful points arise in the course of their work, institutional workers will be encouraged to put them in the form of a question and send them to the Editor, THE HOSPITAL Bureau of Information, 28 & 29 Southampton Street, Strand, W.C. 2, marked "Question Box."

The best questions will be published in due course and our readers will be given the opportunity of answering them. Thus all institutional workers may be able to cooperate with the view of helping the work and smoothing the difficulties of each other. In short, we want the Question Box of the *Institutional Worker* to be freely used by every worker habitually.

### ENQUIRIES AND ANSWERS.

### *THE SICK AND IN NEED.*

### Children's Aid Society.

WE suggest that you communicate with the Secretary, Children's Aid Society, 117 Victoria Street, S.W. The Society rescues, maintains, and trains destitute children.—MRS. C.

### *EMPLOYMENT AND TRAINING.*

### Hospitals in Hong-Kong.

YOU might write to the Secretary of the London Missionary Society, 16 New Bridge Street, London, E.C., with a view to obtaining a nursing post in Hong-Kong. The Society is the governing body of several nursing institutions in Hong-Kong, so that there is just a chance of their being able to help you in this direction.—KATIE.

### Institute Training at Reduced Fee.

IF your husband, being in the Army, was killed during the present war, you will be admitted to the courses of lectures or the examinations of the Royal Sanitary Institute at two-thirds the ordinary fees. This concession also applies to sailors' widows or sailors' or soldiers' wives whose husbands have been permanently disabled during the present war.—WIDOW.

### Anæsthesia Course for Nurses in America.

YES; you are quite right in saying that in America it is possible for a nurse to obtain a course of training in the administration of anæsthetics. The Lakeside Hospital at Cleveland give a six months' course to nurses. If the pupil proves herself to be a competent anæsthetist at the end of that time a certificate of attendance at the course is given.—AMERICAN NURSE.

### *THE HOUSEKEEPERS' DEPARTMENT.*

### Dried Eggs.

"EGALL" is not a substitute for eggs, we understand, but is actually pure dried eggs containing no preservative. It is quite possible to use "Egall" for egg and milk. The producers are the Far Eastern Products Co., Ltd., Birmingham, who make special terms for hospitals.—MATRON.

### Climax Cooking Utensils.

THE process of cooking you refer to was introduced by the Climax Cooker. This process consists of cooking without the articles of food coming into contact with water or steam. The food is cooked in an inner vessel, the outer one (containing the water) being kept at boiling point. In this way none of the nutritious properties are wasted, the meat being cooked in its own vapour, which becomes condensed and forms a rich gravy at the bottom of the vessel. The vessels are so compact that meat and two kinds of vegetables can be cooked at the same time. If you write to the Climax Company, 28 Gray's Inn Road, Holborn, W.C. 1, they will no doubt send you their price-list and full particulars.—H. C. T.

### Aeration Syphon Charger.

THE "Pure Aeration" syphon charger to be obtained of Messrs. L. Lumley and Co., Ltd., The Minories, London, E., is an excellent one. Any number of magnum syphons can be charged over and over again at a cost of one penny per syphon. The price of the charger is 42s. net. The salts, powders, etc., for making soda, seltzer, potass, Apollinaris, and lithia waters can also be obtained of Messrs. Lumley at current market prices.—J. W.

### *MISCELLANEOUS.*

### Repairs to Coolidge Tubes.

IT is now possible to have your Coolidge tube repaired. The British Thomson-Houston Co., Ltd., who hold the British patents of the Coolidge X-Ray Tube, have devised a scheme repairing any of these tubes which may have been damaged or broken. If you write to the Company at Mazda House, 77 Upper Thames Street, London, E.C. 4, they will furnish you with details of their scheme.—X-RAY SPECIALIST.

### An Excellent Adjustable Splint.

THE value of the Williams Universal Extension Splint cannot be too widely known. The war has demonstrated the need for splints which will enable wounds or soft parts to be examined and dressed without interfering with the fractured parts. This splint is especially designed for any typical fracture of the upper limb, and is capable of extensive adjustment. It is made of wire, and so arranged that it can be fixed at any desired angle. Rubber bands are made use of to effect the immobilisation of the limb. The mechanism permits of gradual painless and adequate extension of the broken segments. The splint is an ideal one for treating compound fractures of the upper limbs; it is now placed on the market by Messrs. Allen and Hanburys, Ltd., of 48 Wigmore Street, London, W.—R. B. N.

### Electrically-driven Potato Machine.

IN these days of municipal kitchens and shortage of labour a really reliable machine for preparing potatoes with the least possible waste is more than ever essential. The Imperial Machine Co., of 22 Cricklewood Lane, London, N.W. 2, have placed on the market a first-rate potato machine, which is driven electrically The smallest size has a capacity for 20 lb. of potatoes, which it deals with in one minute. The price of the machine without motor is £34. The cost of electromotors depends upon the current available; they may vary from £10 to £15. Full particulars of this and other useful culinary machinery may be obtained upon application to the company.—M. S.

